# Light-Dependent Electrogenic Activity of Cyanobacteria

**DOI:** 10.1371/journal.pone.0010821

**Published:** 2010-05-25

**Authors:** John M. Pisciotta, Yongjin Zou, Ilia V. Baskakov

**Affiliations:** Center for Biomedical Engineering and Technology, University of Maryland, Baltimore, Maryland, United States of America; University of Wisconsin-Milwaukee, United States of America

## Abstract

**Background:**

Cyanobacteria account for 20–30% of Earth's primary photosynthetic productivity and convert solar energy into biomass-stored chemical energy at the rate of ∼450 TW [1]. These single-cell microorganisms are resilient predecessors of all higher oxygenic phototrophs and can be found in self-sustaining, nitrogen-fixing communities the world over, from Antarctic glaciers to the Sahara desert [2].

**Methodology/Principal Findings:**

Here we show that diverse genera of cyanobacteria including biofilm-forming and pelagic strains have a conserved light-dependent electrogenic activity, i.e. the ability to transfer electrons to their surroundings in response to illumination. Naturally-growing biofilm-forming photosynthetic consortia also displayed light-dependent electrogenic activity, demonstrating that this phenomenon is not limited to individual cultures. Treatment with site-specific inhibitors revealed the electrons originate at the photosynthetic electron transfer chain (P-ETC). Moreover, electrogenic activity was observed upon illumination only with blue or red but not green light confirming that P-ETC is the source of electrons. The yield of electrons harvested by extracellular electron acceptor to photons available for photosynthesis ranged from 0.05% to 0.3%, although the efficiency of electron harvesting likely varies depending on terminal electron acceptor.

**Conclusions/Significance:**

The current study illustrates that cyanobacterial electrogenic activity is an important microbiological conduit of solar energy into the biosphere. The mechanism responsible for electrogenic activity in cyanobacteria appears to be fundamentally different from the one exploited in previously discovered electrogenic bacteria, such as *Geobacter*, where electrons are derived from oxidation of organic compounds and transported via a respiratory electron transfer chain (R-ETC) [3], [4]. The electrogenic pathway of cyanobacteria might be exploited to develop light-sensitive devices or future technologies that convert solar energy into limited amounts of electricity in a self-sustainable, CO_2_-free manner.

## Introduction

Cyanobacteria are of profound biological and biogeochemical importance. Oxygenic photosynthesis carried out by primitive cyanobacteria transformed early Earth's reducing atmosphere into an oxidizing one 2.4 billion years ago and provided for the evolution of complex aerobic life below a protective ozone layer [5]. From the earliest fossil record up to the present, cyanobacteria have continued to thrive, adapt and support higher life by converting solar energy into energy-dense organic compounds. Diverse genera of mat-building and planktonic cyanobacteria are found all over the world, from temperate ponds to some of the driest and most inhospitable environments imaginable, where they serve key ecological roles in energy transduction, nitrogen fixation and as pioneer species [2]. Indeed, these solar-powered prime movers of global nitrogen and carbon cycling probably represent the most important primary producers in the ocean and they colonize barren rock as new land is created through volcanic activity [6]. An astonishing 50% of the planet's biological nitrogen fixation is attributable to tropical and subtropical marine cyanobacteria [7]. In light of their tremendous ecological importance, deeper investigation into the mechanisms by which cyanobacteria convey solar energy to the environment is warranted.

Solar energy reaches the Earth at the rate of the 178,000 TW [8] of which 0.2% to 0.3% is harnessed by cyanobacteria [1]. The amount of energy that passes through cyanobacteria exceeds by more than 25 times the energy demand of human society (∼15 TW); roughly 1,000 times the energy produced by all the nuclear plants on Earth. On a global scale, cyanobacteria fix an estimated 25 Giga tons of carbon from CO_2_ per year into energy dense biomass [1], [9]. The purpose of this study is to investigate whether cyanobacteria possess light-dependent electrogenic activity (i.e. are capable of depositing electrons to the extracellular environment in response to illumination), to test whether the electrogenic activity is generic and to probe the mechanism of the electrogenic response.

## Results

### Diverse cyanobacteria donate electrons to their surroundings

**Figure 1:**
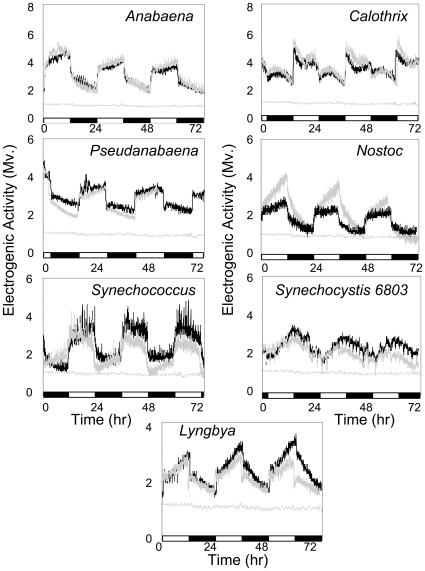
Light-dependent electrogenic activity of diverse cyanobacteria genera. Individual cyanobacterial genera were cultured under photoautotrophic conditions and 24 h illumination cycles (12 h of light/12 h of dark). Electrogenic activity was monitored by recording MFC voltage at 1 KΩ fixed external resistance. Rapid increase in voltage was observed at the beginning of each 12 h light cycle, and rapid drop in voltage was seen at the beginning of each 12 h dark cycle. Electrogenic activity monitored from duplicate MFCs (black and gray lines) revealed reproducible electrogenic profiles for individual genera. The negative controls, where cell voltage was monitored in MFCs loaded only with corresponding media in the absence of cyanobacterial cultures (dotted line), are shown for each experiment. 12 h dark-phases are indicated by black bars along x-axis.

To determine if diverse cyanobacteria possess a conserved ability to transfer electrons to the extracellular environment, we employed an instrumental approach similar to that exploited in microbial fuel cells (MFCs) [10], [11]. In a MFC, the anode functions as the extracellular electron acceptor. The electrogenic activities of ten different genera of cyanobacteria were tested to ascertain whether electrogenic activity is a general cyanobacterial phenomenon. The ten genera were selected to include sheathed, unsheathed, heterocystous, non-heterocystous, filamentous, non-filamentous, biofilm-forming or planktonic cyanobacteria. Cyanobacterial cultures were grown in the anodic chamber of MFCs incubated under photoautotrophic conditions and 24 h illumination cycles consisted of 12 h of light/12 h of dark. At the beginning of illumination, a rapid increase in electrogenic activity was observed from all cyanobacterial genera tested and, particularly, from those that formed biofilms (Fig. 1). The electrogenic activity of all genera declined at the beginning of the daily 12 hours dark phase. A control MFC containing growth media devoid of cyanobacteria lacked any response to light or detectable electrogenic activity (Fig. 1). Similarly, non-photosynthetic control cultures consisting of *Escherichia coli* were insensitive to illumination arguing that the light-dependent electrogenic activity was not due to electrical stimulation of bacteria by light (Figure S1).

**Figure 2:**
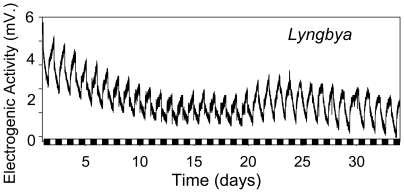
Long-term electrogenic activity. Light-dependent 24 hour oscillations in electrogenic activity could be observed for many days with little variation within each individual light dark cycle, as shown for *Lyngbya*. 12 h dark-phases are indicated by black bars along x-axis.

Each cyanobacterial genus tested revealed a unique light response kinetic profile recorded over the course of consecutive 24 hours cycles (Fig. 1). For each genus these kinetic voltage profiles tended to repeat daily with little variation between days, raising the possibility that the electrogenic activity reflects the peculiar physiological characteristics of individual phototroph cultures. An electrogenic response to light observed from sheathed cyanobacteria (*Phormidium, Nostoc, Spirulina, Anabaena, Lyngbya*) indicated that mucilaginous sheaths do not insulate or prevent electrogenic activity. Observation of electrogenic activity from the non-heterocystous cyanobacterium *Pseudanabaena* or from the non-diazotrophic cyanobacteria *Synechococcus* or *Synechocystis* PCC-6803 indicate that the ability to produce heterocysts or to fix nitrogen, respectively, are not required for electrogenic activity (Fig. 1). Both freshwater and saltwater genera displayed an electrogenic response. Pelagic *Synechocystic* PCC-6803 showed a very weak response to light, suggesting direct contact between cells and the extracellular electron-accepting surface may be the primary means of electron transfer [10]. Light-dependent 24 hour oscillations in electrogenic activity could be observed for many days, as long as MFCs were kept under operation (Fig. 2).

**Figure 3:**
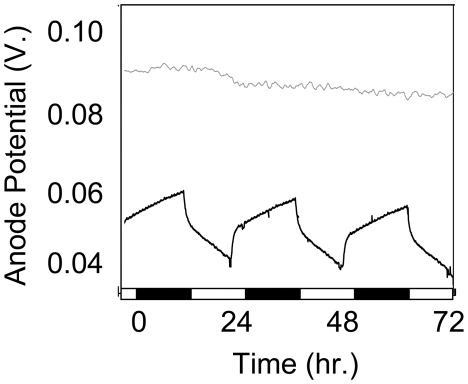
Light dependent oscillations in anode potential. Anode potential in a *Nostoc* containing half MFC (black line) was found to oscillate relative to an Ag/AgCl reference electrode as a function of illumination. An identical anode devoid of biofilm (gray line) was unresponsive to illumination. 12 h dark-phases are indicated by black bars along x-axis.

To test whether electrogenic activity, which was recorded as a difference between an anode and cathode potential in MFC, was indeed attributed to electron flow from the photosynthetic cultures to extracellular electron acceptor, we recorded anode potential during 24 hour illumination cycles (Fig. 3). The anode potential was found to oscillate as a function of illumination cycles providing direct evidence that cyanobacteria deposit electrons onto anode surface when exposed to light. Light-dependent deposition of electrons makes the anode potential less positive compared to the potential in the dark which results in the increase in MFC voltage during the light phase. The control anode devoid of photosynthetic biofilm showed no response to illumination (Fig. 3).

Measurements of dissolved oxygen and pH in cyanobacterial cultures revealed periodic 24 h oscillations that occurred because of light-dependent photosynthetic oxygen evolution (Figure S2). In contrast to the rapid rise of cell voltage observed in direct response to illumination, both dissolved oxygen and pH displayed more gradual increase during 12 h illumination-phase. These results argue that the positive light response was not attributed to change in dissolved oxygen concentration or pH. In fact, an increase in dissolved oxygen or pH creates unfavorable conditions for MFC operation and would normally be expected to reduce MFC voltage during the illumination-phase.

Collectively these results show that light-dependent electrogenic activity is a general physiological feature common to diverse cyanobacteria and that this activity is attributed to electron flow from the cyanobacterial cells to extracellular electron acceptors under illumination.

### Electrogenic activity of a naturally-occurring photosynthetic consortium

**Figure 4:**
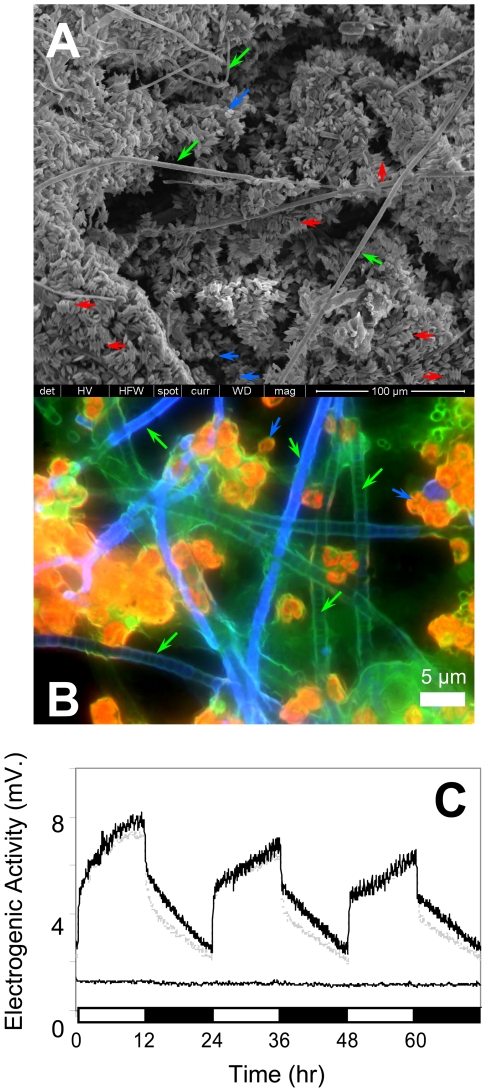
Electrogenic activity of phototrophic biofilm consortium. Scanning electron microscopy (A) and intrinsic fluorescence microscopy (B) images of mixed phototrophic biofilm consortium. Microalgae included individual cells with spherical morphology (blue arrows) and sets of four cells (red arrows). Filamentous and non-filamentous (green arrows) cyanobacteria were apparent in biofilm. (C) Electrogenic activity of duplicate MFCs containing pond biofilm consortia cultivated under photoautotrophic conditions and 24 h illumination cycles (black and gray lines). The negative control represents cell voltage in MFC loaded only with BG11 media in the absence of biofilm (dotted line). 12 h dark-phases are indicated by black bars along x-axis.

In the natural environment, cyanobacteria are often found as a constitutive part of biofilm- or mat-forming mixed consortia composed of cyanobacteria, heterotrophic bacteria and algae. To test whether naturally-occurring photosynthetic biofilm displays electrogenic activity, a phototrophic consortium was collected from a fresh-water pond and cultivated under laboratory photoautotrophic conditions. Upon forming a biofilm (Fig. 4A,B), this photosynthetic consortium displayed electrogenic activity with a strong positive light response indicating that this phenomenon is not limited to the cyanobacterial genera cultivated individually (Fig. 4C). Sequence analysis of bacterial 16S rRNA gene amplicons isolated by denaturing gradient gel electrophoresis (DGGE) identified the bacterial phylotypes phylogenetically most similar to *Phormidium* (95% identity), *Leptolyngbya* (95% identity) and *Pseudanabaena* (96% identity) cyanobacteria; *Sediminibacterium* (93% identity), *Prosthecobacter* (95% identity) and a phylotype most closely related to *Methylococcus* (83% identity) in the mixed consortium of the phototropic biofilm (Table 1). Sequence analysis using 23S rRNA gene primers inclusive for photosynthetic bacterial and eukaryotic microalgae detected 23S rRNA gene amplicons with highest level of identity to those of *Leptolyngbya* (88% identity), *Pseudanabaena* (92% identity) and *Cyanothece* (91% identity) cyanobacteria and the green microalgae *Scenedesmus* (99% identity) (Table 1, Figure S3).

**Table 1:** DGGE analysis of microbial concortia of pond biofilm.

Gene	Closest Phylotype^1^	Identity, %	Genbank #
16S	*Pseudanabaena*	96%	HM122737
16S	*Phormidium*	95%	HM122731
16S	*Leptolyngbya*	95%	HM122733
16S	*Prosthecobacter*	95%	HM122734
16S	*Sediminibacterium*	93%	HM122732
16S	*Methylococcus*	83%	HM122736
23S	*Scenedesmus*	99%	HM122740
23S	*Pseudanabaena* *	92%	HM122735
23S	*Cyanothece*	91%	HM122739
23S	*Leptolyngbya* *	88%	HM122738

1 table ranks the closest phylotypes detected by DGGE using 16S or 23S primer sets. Only the most similar BLAST result for each sequenced band is shown. Genbank accession numbers of closest matches are indicated.

*asterisk indicates genera that were detected by both 23S and 16S primer sets.

### Inhibitor studies indicate H_2_O is electron source

**Figure 5:**
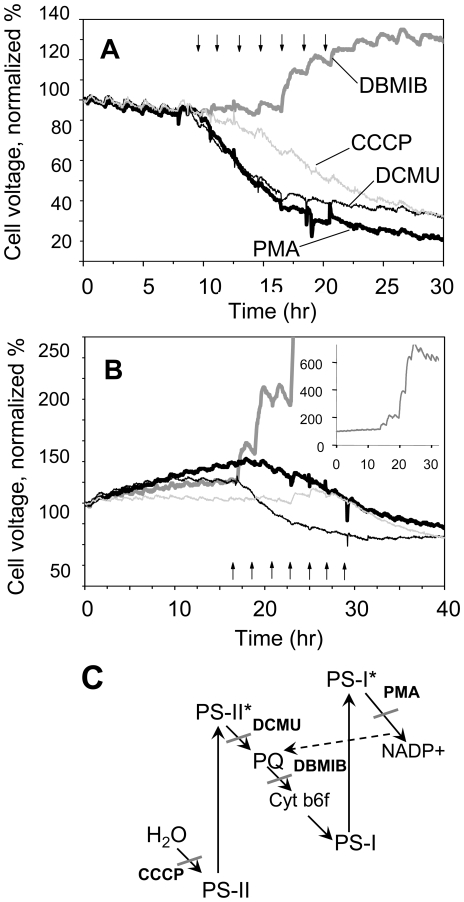
Effect of site-specific inhibitors on electrogenic activity. The site-specific P-ETC inhibitors were added to *Lyngbya* (A) or *Nostoc* (B) at 2 hours intervals at the following cumulatively increasing concentrations: 1, 5, 10, 25, 50, 75 and 100 µM for DCMU (thin black lines), DBMIB (thick gray lines) and PMA (thick black lines); or 5, 10, 25, 50, 100, 150 and 200 µM for CCCP (thin gray lines) as indicated by arrows. The electrogenic activity was monitored under constant illumination and at 1 KΩ fixed external resistance. The effect of DBMIB on electrogenic activity of *Nostoc* is shown in the inset. The experiments were repeated three times giving consistent results. (C) Schematic diagram showing P-ETC and sites targeted by inhibitors. Cyclic electron transfer (Q-cycle) is indicated by dashed line.

The immediate, rapid onset of the positive response to illumination is consistent with the hypothesis that the photosynthetic electron transfer chain (P-ETC) is the source of electrons responsible for electrogenic activity. To test this hypothesis, four site-specific inhibitors of P-ETC components were administered and their effect on electrogenic activity was recorded using two individual cultures, *Nostoc* and *Lyngbya*. *Nostoc* and *Lyngbya* were selected for more detailed studies because they provided relatively strong electrogenic light responses, grew rapidly in anode chambers of MFCs and also formed excellent anode biofilms. Both inhibitors that target photosystem II (PS-II), carbonyl cyanide m-chlorophenylhydrazone (CCCP) and 3-(3,4-dichloro-phenyl)-1,1-dimethylurea (DCMU), and an inhibitor of photosystem I (PS-I), phenylmercuric acetate (PMA), reduced the electrogenic activity in illuminated *Nostoc* and *Lyngbya* (Fig. 5A,B). In contrast, 2,5-dibromo-3-methyl-6-isopropylbenzoquinone (DBMIB), which targets cytochrome b6f, substantially increased the electron discharge to the extracellular acceptor in both cyanobacteria. These results support the hypothesis that the electrons responsible for the electrogenic activity originate from PS-II-mediated biophotolysis of water and that plastoquinone (PQ), located between the targets of DCMU and DBMIB, plays a central role in electron transport from the P-ETC to the extracellular environment (Fig. 5C). Inhibition of electrogenic activity by PMA was due to interference of PMA with the Q-cycle of electron transfer that feeds electrons from ferredoxin back to PQ [12], [13] (Fig. 5C).

To confirm that electrons originate from PS-II, we used an artificial quinone tetramethyl-*p*-benzoquinon (duroquinone), which is known to compete with DCMU for binding to the Q_B_ site on PS-II. Unlike DCMU, however, duroquinone is able to efficiently shuttle electrons from PS-II to PS-I by simulating the function of PQ [14]. Therefore, we were interested in testing whether duroquinone could restore electrogenic activity after inhibition by DCMU. Following partial inhibition of electrogenic activity by 10 µM DCMU, treatment with 25 µM duroquinone fully restored electrogenic activity (Figure S4). Subsequent treatment with additional 75 µM duroquinone was found to boost the electrogenic activity even further (Figure S4). Therefore, duroquinone not only successfully competed with DCMU, but also substituted PQ as an electron transporter. This result is consistent with previous studies reporting that duroquinone is capable of mimicking the properties of PQ, and it supports the notion that the electrons donated to the environment are derived from water and transit via the P-ETC.

### Electrogenic activity is driven by blue or red but not green light

**Figure 6:**
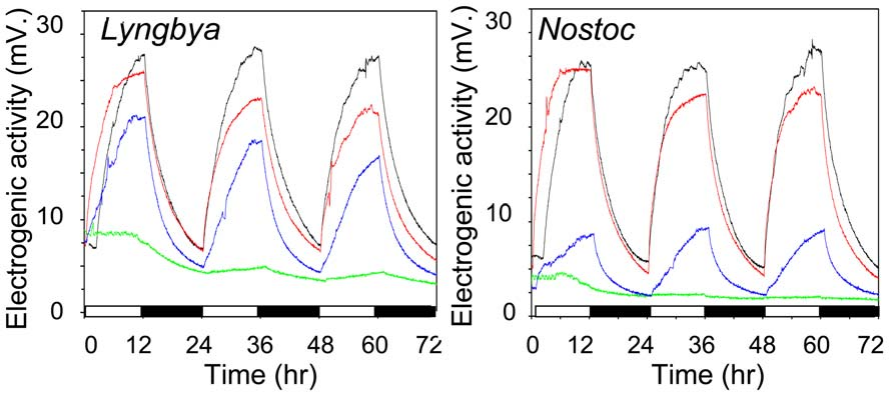
Electrogenic activity under light of different colors. Electrogenic activity was recorded from *Lyngbya* (left panel) or *Nostoc* (right panel) cultured under 24 h illumination cycles (12 h of light/12 h of dark) under red light (red lines), blue light (blue lines), or green light (green lines) of equal intensities. Electrogenic activity under standard white light source of the intensity equal to that of individual colors is provided as a reference (black lines). These experiments were performed using improved anodes that were constructed using nanostructured polypyrrole as previously described [15]. Because of improvements in anode properties, the amplitude of the light response was substantially higher that those reported for the same cultures in Fig. 1. Because nanostructured polypyrrole has properties of a capacitor, the shape of light response curves changed (more gradual increase in response to light). 12 h dark-phases are indicated by black bars along x-axis.

In cyanobacteria, the light harvesting system consists of *chlorophyll a* (two excitation maxima at 440 and 680 nm) and phycobilisomes that include two major pigments: *phycocyanin* (an excitation maximum between 595–640 nm) and *allophycocyanin* (an excitation maximum between 650–655 nm). As a result of the composition of light harvesting systems, the photosynthic reactions in cyanobacteria are primarily driven by red and blue but not green light, a phenomenon known as a “green gap”. If electrons derive from the P-ETC then electrogenic activity should only be observed under photsynthetically active blue or red light but not green light. As expected, the electrogenic activity was observed under red or blue light but not green light in both cyanobacterial cultures (*Nostoc* and *Lyngbya*) used in this experiment (Fig. 6). The electrogenic response was found to be higher under red than blue light. This result is consistent with the predominant role of phycobilisomes in light harvesting in cyanobacteria. Phycobilisomes are pigment-protein complexes composed of *phycocyanins* and *allophycocyanins* which absorb red light.

### Estimation of the yield of the electrogenic activity

**Figure 7:**
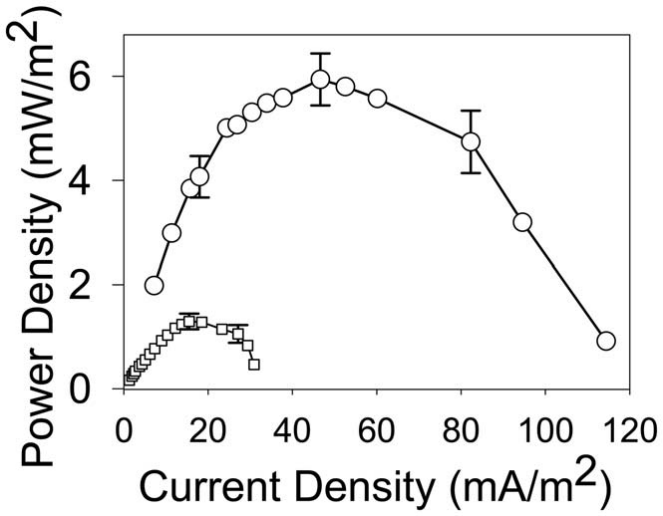
Effect of anode material on electron harvesting. Power density curves (normalized by the cathode surface area  = 9.6 cm^2^) measured for MFC with mixed photosynthetic biofilm consortium formed on anode coated with polypyrrole (□) or nanostructured polypyrrole (○). Error bars represent standard deviation based on three independent experiments.

By measuring the amount of electrons passing through the electrical circuits of MFCs under well controlled illumination conditions, it was possible to estimate the apparent yield of electrons harvested by the MFC anodes to the photons available for photosynthesis for each culture (Table 2). The yield of harvested electron varied from 0.051% (for *Leptolyngbya*) to 0.3% (for pond consortium). Although all yields were within one order of magnitude of each other these yields should be regarded as semi-quantitative since technical difficulties precluded accurate estimation of the biofilm biomass in individual cultures. While, in general, the yield of electron discharge appeared to be quite low, we found that it could be substantially increased by improving the design of the electron-harvesting system [15]. For instance, replacing polypyrrole, an electrically conductive polymer that helps to harvest electrons, with a nanostructured polypyrrole on the anode surface increased the electrical power outputs by ∼4.5-fold (Fig. 7). Furthermore, the yield of electrogenic activity appears to be underestimated because dissolved oxygen can react with electrons and protons to form water thereby interfering with electron harvesting by extracellular acceptors. Therefore, the yield of electron discharge depends not only on the physiology of the particular genus, but also on the immediate environment and the chemical composition and physical structure of the substrate that supports biofilm formation.

**Table 2:** Electrogenic yield of diverse cyanobacteria genera and mixed pond consortium.

Culture	Yield, % 1
Pond consortium	0.304±0.009
*Calothrix*	0.265±0.006
*Pseudoanabaena*	0.165±0.008
*Synechococcus*	0.155±0.006
*Ananbaena*	0.149±0.015
*Phormidium*	0.149±0.015
*Nostoc*	0.136±0.013
*Lyngbya*	0.130±0.016
*Spirulina*	0.099±0.09
*Synechocystis*	0.075±.008
*Leptolyngbya*	0.051±0.015

1 The yield is shown as a mean of three 24 h illumination cycles with a standard deviation.

## Discussion

The current studies show that cyanobacteria exhibit light-dependent electrogenic activity and that this activity is conserved among diverse genera. Previously, only chemotrophic bacteria were found to display intrinsic electrogenic activity [4], [16]. In electrogenic chemotrophs such as *Geobacter sulfurreductens*, electrons are derived from oxidation of organic compounds and transported via a respiratory electron transfer chain (R-ETC) to extracellular terminal electron acceptors [3]. In *Geobacter*, the electron transfer to the environment was shown to be mediated by a diverse group of c-type cytochromes and possibly involved electrically-conductive microbial nanowires [17]–[19]. In previous studies, exogenous electron mediators such as 2-hydroxy-1, 4-naphtoquinone were employed for recording electrogenic activity from cyanobacterial cultures [20], [21]. Because this synthetic quinone is capable of intercepting electrons at numerous sites along the R-ETC and the P-ETC, it was difficult to determine whether cyanobacteria possess natural mechanism(s) for electron discharge to the extracellular environment and, if they do, whether R-ETC or P-ETC is the source of electrons. The present work expands our basic understanding of bacterial electrogenesis and demonstrates that diverse cyanobacteria exhibit light-dependent electrogenic activity. In contrast to electrogenic chemotrophs, cyanobacterial electrogenic activity was observed in the absence of any exogenous organic fuel and was driven entirely by the energy of light.

We show that the P-ETC is the source of electrons and that PQ plays a central role in electron flow from cyanobacteria to the extracellular environment; in this case the MFC anode. In cyanobacteria PQ is known to be present in substantial amounts in both cellular and thylakoid membranes [9], [22]. Inhibition of PS-II by DCMU or CCCP rapidly reduced the light-dependent electrogenic activity in *Nostoc* and *Lyngbya*, providing support that electrons originate from PS-II-mediated photolysis of water (Fig. 5). CCCP inhibits electron flow through PS-II but can also uncouple the electron-transport-dependent ATP synthesis by inactivating ATPase [23]. DCMU, however, is known to be a specific PS-II inhibitor that blocks binding of PQ to PS-II [24], [25]. Blocking cytochrome b6f activity with DBMIB resulted in a significant increase in electrogenic activity. DBMIB is a PQ analogue that binds cytochrome b6f and prevents electron transfer from reduced PQ to cytochrome b6f [22], [26], [27]. The opposing effects of DCMU and DBMIB indicate electrons probably exit P-ETC downstream of PS-II and upstream of cytochrome b6f. PQ is the only P-ETC component located between these two P-ETC complexes. Furthermore, the rescue of DCMU-induced inhibition by duroquinone, which has a very high capacity as a PS-II electron acceptor and can shuttle water-derived electrons from PS-II [14], strongly support the mechanism that electrons originate from PS-II-mediated photolysis of water. Inhibition of electrogenic activity by PMA was likely due to interference of PMA with the Q-cycle of electron transfer [12], [13]. In the Q-cycle, electrons are transported from PS-I to ferredoxin, and then fed back to PS-I through PQ and cytochrome b6f complex (Fig. 5C). P-ETC inhibitors acted in a similar fashion in both *Nostoc* and *Lyngbya* implying that the mechanism responsible for electrogenic activity is conserved. Although the biological function of electrogenic activity in cyanobacteria is not yet clear, it is tempting to speculate that it could help regulate the redox state of PQ by shedding excess energy to the environment when PQ becomes over reduced in high light. Future studies will examine this possibility.

An independent approach that employed lights of different color strongly supported a direct link between electrogenic activity and the cyanobacterial photosystem. The fact that electrogenic response was observed only under red or blue light is consistent with the notion that the electrons are donated to the extracellular environment via the P-ETC (Fig. 6). Green light, which is not absorbed by light-harvesting pigments, was unable to induce a positive light response. The electrogenic response under red light was found to be almost as high as that under the reference white light. This result is consistent with the fact that red light absorbing phycobilisomes play a major role in light harvesting in cyanobacteria. The differences in the ratio of photosynthetic pigments in individual genera could explain the different amplitude of the electrogenic response between *Nostoc* and *Lyngbya* under the blue light (Fig. 6).

While it is tempting to speculate electrogenic activity may provide a means of shedding excess energy under intense light, other functions, such as a role in carbon fixation, might also be possible. For efficient carbon fixation, cyanobacteria actively import HCO_3_^−^ using membrane-spanning transporters [28]. Interestingly, some cyanobacteria that lack systems for CO_2_ uptake still possess HCO_3_^−^ uptake systems, indicating HCO_3_^−^ is the preferred inorganic source for carbon fixation [29]. One of the reason for preferential HCO_3_^−^ uptake is that the HCO_3_^−^ anion is much less membrane permeable than CO_2_, which readily diffuse out of cells. Once inside the cell, cytosol accumulated HCO_3_^−^ is converted to CO_2_ for carbon fixation by carboxysomal carbonic anhydrase (HCO_3_^−^+H^+^>H_2_CO_3_>CO_2_+H_2_O). In the present study, pH and dissolved oxygen rose to high levels after illumination of diverse genera (Figure S3) and a voltage spike was observed whenever the light turned on. One possibility, to be followed up on by future studies, is that electrogenic activity could relate to the carbon concentrating mechanism and intracellular filling of the inorganic carbon reserves upon illumination. Indeed, previous researchers have suggested CO_2_ entering cells can become trapped intracellularly by conversion to HCO_3_^−^ through a P-ETC associated pathway [29]. When pure CO_2_ was administered to MFCs electrogenic activity dropped sharply before rebounding within a few minutes indicating electron donation to the extracellular environment had been temporarily interrupted (data not shown). In the future, MFCs can serve as tools to study electrogenic activity as it may relate to the carbon concentrating mechanism.

In anaerobic chemotrophs, electrogenic activity is a constituent part of bacterial respiration, whereas in cyanobacteria we speculate electrogenic activity might help cells adapt to unfavorable light conditions. To cope with the adverse effects of intense sunlight cyanobacteria have evolved several protective mechanisms [30]. To prevent ultraviolet damage mat-building cyanobacteria synthesize sunscreens such as scytonemin which accumulates in trichome sheaths [31]. Inducible non-photochemical quenching can limit the solar energy conveyed to PS-II by increasing the amount of light dissipated as heat [32]–[34]. Negative phototaxis triggered by increasing ROS concentration allows some motile cyanobacteria to shield themselves from intense sunlight and gain exposure to optimal intensity light by burrowing downward [35], [36]. Despite this broad diversity, the currently known adaptive responses may not be effective against rapid fluctuations in light intensity, suggesting other protective mechanisms could also exist. Considering that light-dependent electrogenic activity is conserved among diverse genera of cyanobacteria, one possibly is that electrogenic activity might serve as a protective mechanism to adverse environmental conditions, such as rapid fluctuation or high intensity of sunlight.

Our study found that a cyanobacteria-containing biofilm consortium exhibited electrogenic activity in a manner similar to that of individual cultures (Fig. 4). DGGE analysis of 16S and 23S rRNA genes indicated the presence of several mat building cyanobacteria. Gene sequence analysis of the electrogenic pond biofilm indicated these cyanobacteria were phylogenetically most similar to *Phormidium*, *Leptolyngba* and *Pseudanabaena*. A green algae and chemotrophic bacteria were also indicated. Minor microbial constituents of the biofilm may not have been detected by DGGE analysis. Nevertheless, each of these three cyanobacterial genera showed light-dependent electrogenic activity when grown as individual cultures (Fig. 1).

Cyanobacteria are the most successful mat-building organisms. They form the topmost, aerobic layer of microbial mats where access to light, atmospheric CO_2_ and N_2_ is greatest [37]. A layer of oxidized iron may separate the cyanobacterial oxygenic layer from a lower anoxygenic layer composed of purple sulfur and green sulfur bacteria. In marine mats, anaerobic sulfate-reducing bacteria can be found throughout the mat below the top layer of cyanobacteria. Sulfate-reducing bacteria play a major role in decomposing organic materials produced by cyanobacteria. The joint metabolic activity of microorganisms in mats results in steep gradients of light, oxygen, carbon dioxide, pH, and redox potential [37]. One untested possibility is that rather than merely shedding excess solar energy to the abiotic environment, cyanobacteria might donate excess water-derived electrons to biofilm symbionts.

The ability of cyanobacteria to donate electrons directly to the extracellular environment (i.e. anode) was illustrated by the decreasing anode potential observed when a *Nostoc* containing half MFC was exposed to light (Fig. 3). An anode without cyanobacteria displayed no such response, indicating this phenomenon is mediated by cyanobacterial cells. The relatively low yield of electron harvesting by extracellular acceptors observed in our experiments was in part due to high amounts of dissolved oxygen present at concentrations substantially exceeding those found in natural cyanobacterial mats. However, the yield of electron harvesting could be improved by as much as 4.5-fold simply by changing the nanostructure of the anode surface. In natural cyanobacterial mats, the yield of electron discharge is likely to be variable, depending on the chemical environment, the physical properties of electron acceptors, the intensity of solar radiation, the concentration of dissolved oxygen and other factors. Even if a very modest yield is used for estimating the average rate of electron discharge, the transfer of solar energy to the environment via cyanobacterial electrogenic pathway could proceed at the rate of ∼9 TW on a global scale. Therefore, the electrogenic pathway appears to be an important microbiological conduit of solar energy into the biosphere and could have significant impact on a global scale. Anticipated applications of the electrogenic activity of cyanobacteria described here might be the biological conversion of solar energy to electrical energy or self-sustainable light sensors. Although at present the conversion yield is quite low, future studies on improvements in anode design, genetic manipulations of P-ETC or strain selection will answer the question of whether self-sustainable, CO_2_ free technologies based on the light-dependent electrogenic activity of cyanobacteria are feasible.

## Materials and Methods

### Cultures

Ten genera of cyanobacteria (Table 1) were obtained from the CCMP (Center for Culture of Marine Phytoplankton, West Boothbay Harbor, ME). All cultures were grown at 24.0°C under a cool white fluorescent light source (light intensity ∼100 lux, color temperature 6500 K) operated under a 24 hour illumination cycles (12 h of light/12 h of dark). Marine cultures were grown in F2 medium (NaNO_3_, NaH_2_PO_4_•H_2_O, trace metals, vitamins B1, H and B12) and freshwater cyanobacteria were grown in modified DY-V medium (MgSO_4_, KCl, NH_4_Cl, NaNO_3_, H_3_BO_3_, CaCl_2_, trace metals and vitamins B1, H and B12) that lacked Na_2_ β-glycerophosphate and MES, prepared from CCMP culture media kits as described in manufacture's instructions. A natural biofilm-forming photosynthetic consortia was collected from a pond in Columbia, MD and adapted to modified BG-11 medium that lacked citrate as previously described [10]. *E. coli* grown in LB media or LB media diluted 1∶1 in F2 media were tested under the same 12∶12 hr light cycling conditions as a non-photosynthetic control cultures.

### Electrogenic activity

For recording electrogenic activity, individual cyanobacterial cultures or mixed biofilm consortia were seeded into the transparent anodic chambers (150 mL) of photosynthetic MFCs. MFCs were constructed as previously described and equipped with carbon paint anodes coated with electrically conductive polypyrrole [10]. Following a two weeks acclimation/growth period, the electrogenic activity was monitored by recording MFC voltage at 1 KΩ fixed external resistance using a digital data acquisition system (PCI-6280, National Instruments, Austin, TX) equipped with LabVIEW software (National Instruments). The electrogenic activity was monitored over the course of up to 30 consecutive days for each culture. The power curves were calculated from the polarization curves as previously described [11]. For recording the polarization curve, MFCs were stabilized at an open circuit potential, then the voltage was measured at variable external resistances (from 100 KΩ to 10 Ω) after stabilization for 15 min at each resistance, and the current was calculated using Ohm's Law.

### Testing of site-specific inhibitors

After seeding and acclimation as described above, the electrogenic activity was monitored for 5 days for both *Nostoc* and *Lyngbya* as described above. When the electrogenic activity reached a plateau on the day 6th, the cultures were dosed at 2 hours intervals with cumulatively increasing concentrations of the following inhibitors: carbonyl cyanide m-chlorophenylhrazone (CCCP), 3-(3,4-dichloro-phenyl)-1,1-dimethylurea (DCMU), 2,5-dibromo-3-methyl-6-isopropylbenzoquinone (DBMIB) or phenylmercuric acetate (PMA). DBMIB, DCMU, and PMA were added at the following concentrations: 1, 5, 10, 25, 50, 75, 100 µM; and CCCP was added at the following concentrations: 5, 10, 25, 50, 100, 150 and 200 µM. The effect of inhibitors on electrogenic activity was monitored by recording MFC voltage at 1 KΩ fixed external resistance as described above under constant illumination.

### Assessment of electrogenic yield

The electrogenic yield for each genera was calculated as the ratio of electrons passing through the electrical circuit of the MFC to the total electrons associated with the known photon flux illuminating the 50 cm^2^ anode surface. Normal 12/12 hr light-dark cycling was employed. Illumination with 100 lux cool white fluorescent light equates to a surface photon energy input of 1.4 µmoles/m^2^/s. To calculate the number of anode electrons harvested, the recorded voltage curves for each genera were converted to amps of current using Ohm's Law (I = V/R). This reveals the number of Coulombs collected per second since one Amp is defined as one Coulomb per sec (one Coulomb  = 6.241×10^18^ electrons, i.e. 0.0103 mM). Integration of the area under the Amp curve after subtraction of background reveals the total number of anode electrons collected. The mean value and standard deviation were calculated using the data obtained for three 24 h illumination cycles for each individual culture. Because of the technical difficulties in quantitating biofilm formed on anode, the data were not normalized per number of cyanobacterial cells. Instead, different genera grown to approximately equivalent cell densities in MFC anode chambers were used for estimating the yield.

### Microscopy imaging of mixed biofilm consortia

The mixed biofilm was transferred from the MFC anode to a glass cover slip via sterile pipette tip (Fisher Scientific Co., Pittsburgh, PA), and immediately imaged on an Nikon Eclipse TE200-U inverted fluorescence microscope (Nikon Inc., Melville, NY), equipped with an X-Cite illumination system (EXFO Photonics Solutions Inc., Quebec, CA) connected by fiber optic cable to a 1.3 aperture Plan flour ×150 numerical aperture ×60 objective. Digital images were taken in three channels using a 12-bit Coolsnap HQ CCD camera (Photometrics, Surrey, CA) and the following filter cubes: EN-GFP-HQLP (Nikon Inc. Melville, NY, excitation at 470±20 nm, long-pass emission above 500 nm), FITC C31001 (Chroma Technology Corp., Rockingham, VT, excitation at 480±15 nm and emission at 535±20 nm), and Eth-Bro C31008 (Chroma Technology Corp., excitation at 540±12.5 nm and emission at 605±27.5 nm). The exposure times were automatically optimized using the V++ version 4.0 software (Digital Optics Ltd., Auckland, NZ). The fluorescent channels were merged using WCIF Image-J (National Institutes of Health, Bethesda, MD).

For scanning electron microscopy (SEM) imaging, a small piece of the anode with a well-established biofilm was fixed in a phosphate buffer containing 2% glutaraldehyde for 1 h following by washing with deionized water for 10–20 min. The sample was dehydrated by gradually increasing the acetone concentration from 20%–100% and then dried in CO_2_. The samples were coated with Au and imaged using a JEOL 4000 electron microscope.

### DGGE analysis of mixed biofilm-forming consortia

The biofilm cells were scraped from the anode surface with a sterile pipette and lysed by freezing/thawing in liquid nitrogen followed by shaking with 0.1 mm glass beads for 2 min in a mini-bead beater (Biospec Products, Bartlesville OK). Total DNA was extracted with the GeneElute plant DNA kit (Sigma-Aldrich Corp. St. Louis, MO) and DNA integrity was checked by agarose gel electrophoresis. PCR amplification using cyanobacteria-specific, bacteria-specific, or phototroph-specific primers equipped with a 40 bp GC clamp on the 5′ end (Table S1, Text S1) was carried out using Maxima hot-start Taq DNA polymerase according to the manufactures' instructions (Fermentas Life Sciences, Burlington, CA). All PCR reactions were carried out in 50 ul reactions volumes using 5 ul of template DNA in DI H_2_O. For the 23 s primer set (p23SrV_f1 and p23SrV_r1), PCR conditions included an initial 5 minute denaturation step at 94.0°C, followed by 35 cycles each consisting of denaturation at 94.0°C for 20 seconds, annealing at 55.0°C for 30 seconds and extension at 72.0°C for 30 seconds. A final extension step was carried out at 72.0°C for 10 minutes.

For the 1401 bacterial primer set (f968-GC and R1401a/b) an initial 5 minute 95.0°C denaturation step was followed by 35 total cycles, each cycle initiated by a 1 minute denaturation step at 95.0°C. For the first ten (touchdown) cycles, the 1 minute annealing temperature was gradually reduced by 0.5°C per cycle from an initial annealing temperature of 60.0°C for down to 55.0°C. For all cycles elongation was at 72.0°C for 2 minutes. For the final 25 cycles 55.0°C was used as the annealing temperature. A final 30 minute elongation was carried out at 72.0°C after the last cycle.

For the 50f cyanobacteria specific primer set (50f and r718a), initial denaturation was 15 minutes at 95.0°C was followed by 35 cycles of 95.0°C denaturation for 1 minute, 55.0°C annealing for 1 minute, and 72.0°C elongation for 1 minute. The final extension step was 7 minutes at 72.0°C. Inclusion of a GC clamp was required for proper DGGE band separation. The sequence of the new 50f primer is 5′AACACATGCAAGTCGAACG-3′.

DGGE of the PCR amplicons was then run using a DGGE-#1001 system (CBS Scientific Del Mar, CA) at 75 V for 16 h in 0.75 mm 6% polyacrylamide gels cast with a 40–65% linear gradient of denaturant (Deiz and Bergman, 2007) in 1xTAE running buffer. Gels were washed in MilliQ water for 10 minutes and then stained 30 min with SYBR Gold nucleic acid stain (Molecular Probes Inc. Eugene, OR). DGGE bands were imaged under a UV transilluminator and excised with sterile razor blades. They were placed in 50 µl of DNAse free H_2_O for overnight incubation at 4.0°C. PCR re-amplification of this template using the primers devoid of the GC clamp was carried out in preparation for sequencing. Reamplified DNA was subjected to agarose gel electrophoresis, the bands were excised and then purified with the MinElute Gel Extraction Kit (Quiagen Co., Venlo, Netherlands). Purified DNA from each band was then sequenced on an ABI 3130 XL Genetic Analyzer (Applied Biosystems, Carlsbad, CA). Sequences were compared to those in the public databases by BLAST search to identify the major biofilm constituents.

### Electrogenic response to different colors of light

*Nostoc* or *Lyngbya* were first cultured under 100 LUX cool white fluorescent light as described above. Once the biofilms were established on anodes, MFCs were transferred to a light box equipped with blue (λ_max_ = 463 nm, half-bandwidth  = 22 nm), green (λ_max_ = 528 nm, half-bandwidth  = 40 nm) and red (λ_max_ = 642 nm, half-bandwidth  = 19 nm) LED light source (Photon System Instruments SL-3500, Brno, Czech Republic). Electrogenic activity of cultures were then monitored under 24 h illumination cycles under light of the three individual colors for 3 consecutive days for each color. The intensity of light for each individual color was adjusted to 100 LUX using Photon System Instruments control unit.

### Recording anode potential

To record the light-dependent oscillations in anode potential following illumination, half MFCs that lacked cathode were constructed. A KCl saturated Ag/AgCl reference electrode was placed into half MFC at distance of 0.5 cm to the anode surface. The anode potential versus the reference electrode was recorded using the digital data acquisition system (PCI-6280, National Instruments, Austin, TX).

### pH and Dissolved Oxygen measurements

Dissolved oxygen (DO) and pH were measured in anode chambers every 10 minutes with an Orion 4 star pH/DO meter (Thermo Orion, Beverly, MA), Ross Ultra glass pH electrode, DO probe (model 081010MD, Thermo Orion) and Star Navigator Plus software (Thermo Orion).

## Supporting Information

Figure S1 A non-photosynthetic electrogenic bacterium lacks light dependent electrogenic response. 1×10^9^ *E. coli* cells were seeded into MFCs containing LB media (A), or LB media diluted 1∶1 in F2 media (B), and MFCs were operated for 12 days at 25°C under the same 12∶12 hr light cycling conditions used to test cyanobacteria. Consistent with the previous studies of *E. coli* [4], [5], MFCs seeded with *E. coli* showed electrogenic activity, however, no oscillating, light dependent electrogenic response was detected from *E. coli*. 12 h dark-phases are indicated by black bars along x-axis.
(0.37 MB TIF)

Figure S2 Dynamics of Dissolved Oxygen (black lines, left axis) and pH (gray lines, right axis) during three consecutive illumination cycles monitored in MFC anodic chambers with *Lyngbya* (A), *Nostoc* (B), *Synechococcus* (C), *Leptolyngbya* (D), or *Calothrix* (E). 12 h dark phases are indicated by black bars along x-axis.
(0.90 MB TIF)

Figure S3 DGGE analysis of mixed biofilm consortia. DGGE analysis of DNA extracted from mixed biofilm consortia and amplified with bacterial 16S rRNA gene specific primers (f968-GC and R1401a/b) (A) or phototroph 23S rRNA gene specific primers (p23SrV_f1 and p23SrV_r1) (B). Bands were excised, purified, re-amplified using the primers f968 and R1401a/b or p23SrV_f1 p23SrV_r1 and sequenced. BLAST searches suggested *Phormidium* (a1, a5), *Leptolyngbya*, (a6, b2), *Pseudanabaena* (b1 and b3) and *Cyanothece* (b4) cyanobacteria. The chemotrophic bacteria phylogenetically most similar to *Sediminibactetium* (a2), *Prosthecobacter* (a3, a4) and *Methylococcus* (a7) were also detected.
(0.22 MB TIF)

Figure S4 Duroquinone rescues DCMU-inhibited electrogenic activity. Administration of 10 µM DCMU (black arrows) to *Lyngbya* (top) or *Nostoc* (bottom) containing MFCs partly inhibited electrogenic activity under constant 100 lux light. After DCMU-associated inhibition of electrogenic activity reached a stable plateau, treatement with 25 µM duroquinone (grey arrows) fully restored electrogenic. Subsequent treatment with additional 75 µM duroquinone (second grey arrows) boosted the electrogenic activity.
(0.41 MB TIF)

Text S1 (0.04 MB DOC)
