# Supplementary figures and images for: Light-Dependent Electrogenic Activity of Cyanobacteria
Source: PLoS One. 2010 May 25;5(5):e10821. doi: 10.1371/journal.pone.0010821 (PMC2876029; doi:10.1371/journal.pone.0010821)

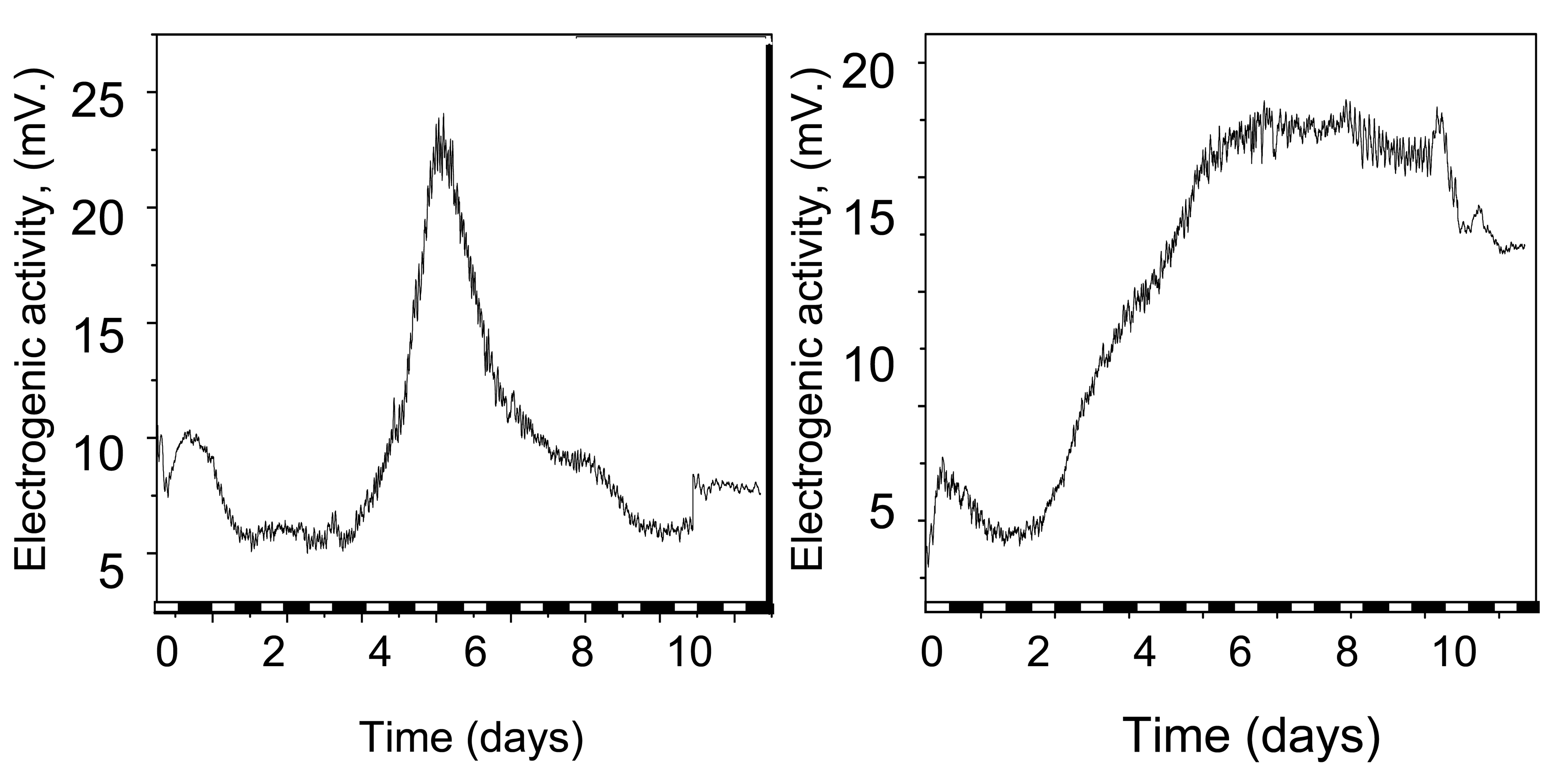

Supplement: Figure S1 — A non-photosynthetic electrogenic bacterium lacks light dependent electrogenic response. 1×10^9^ E. coli cells were seeded into MFCs containing LB media (A), or LB media diluted 1∶1 in F2 media (B), and MFCs were operated for 12 days at 25°C under the same 12∶12 hr light cycling conditions used to test cyanobacteria. Consistent with the previous studies of E. coli [4], [5], MFCs seeded with E. coli showed electrogenic activity, however, no oscillating, light dependent electrogenic response was detected from E. coli. 12 h dark-phases are indicated by black bars along x-axis. (0.37 MB TIF) [file pone.0010821.s001.tif]

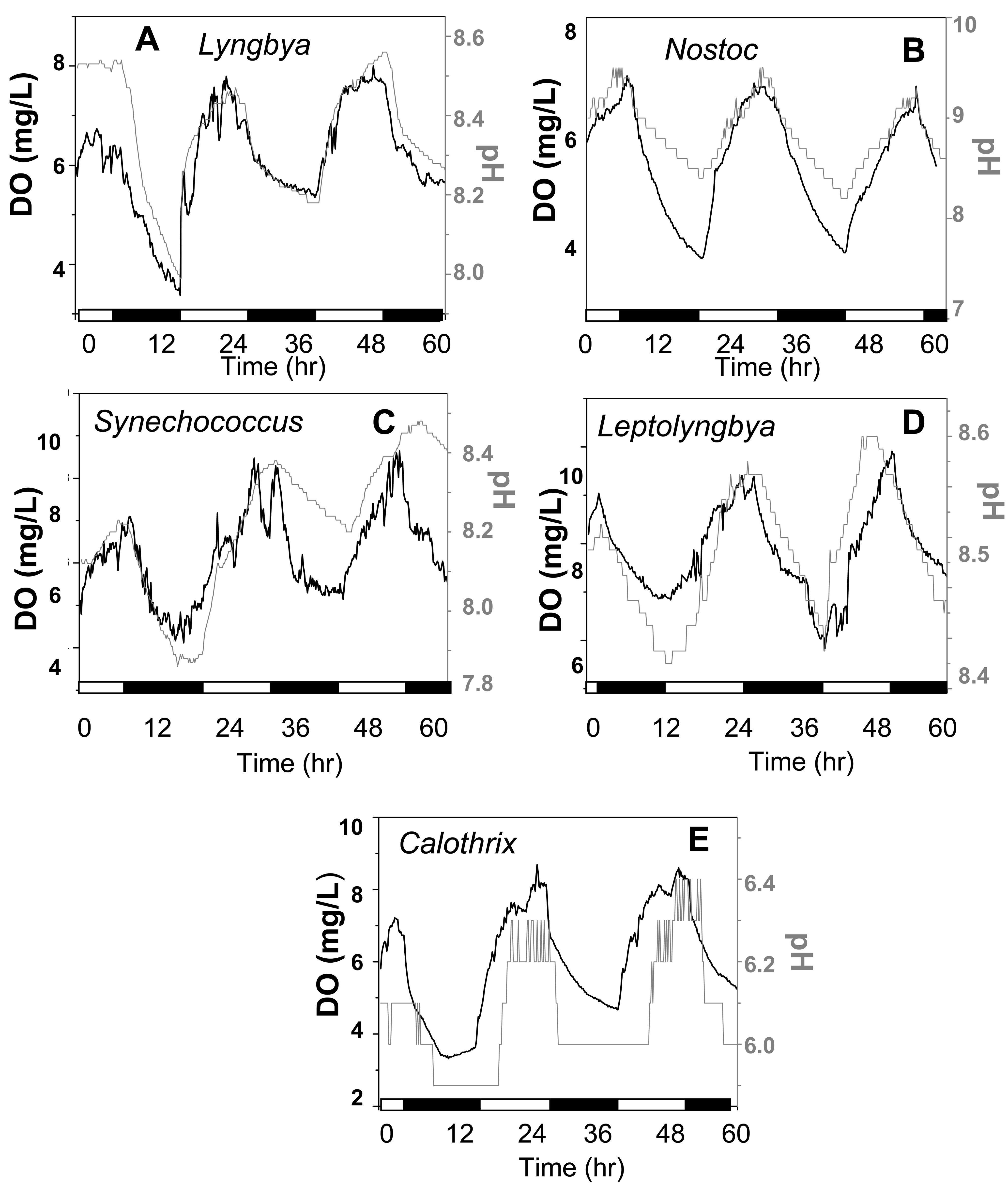

Supplement: Figure S2 — Dynamics of Dissolved Oxygen (black lines, left axis) and pH (gray lines, right axis) during three consecutive illumination cycles monitored in MFC anodic chambers with Lyngbya (A), Nostoc (B), Synechococcus (C), Leptolyngbya (D), or Calothrix (E). 12 h dark phases are indicated by black bars along x-axis. (0.90 MB TIF) [file pone.0010821.s002.tif]

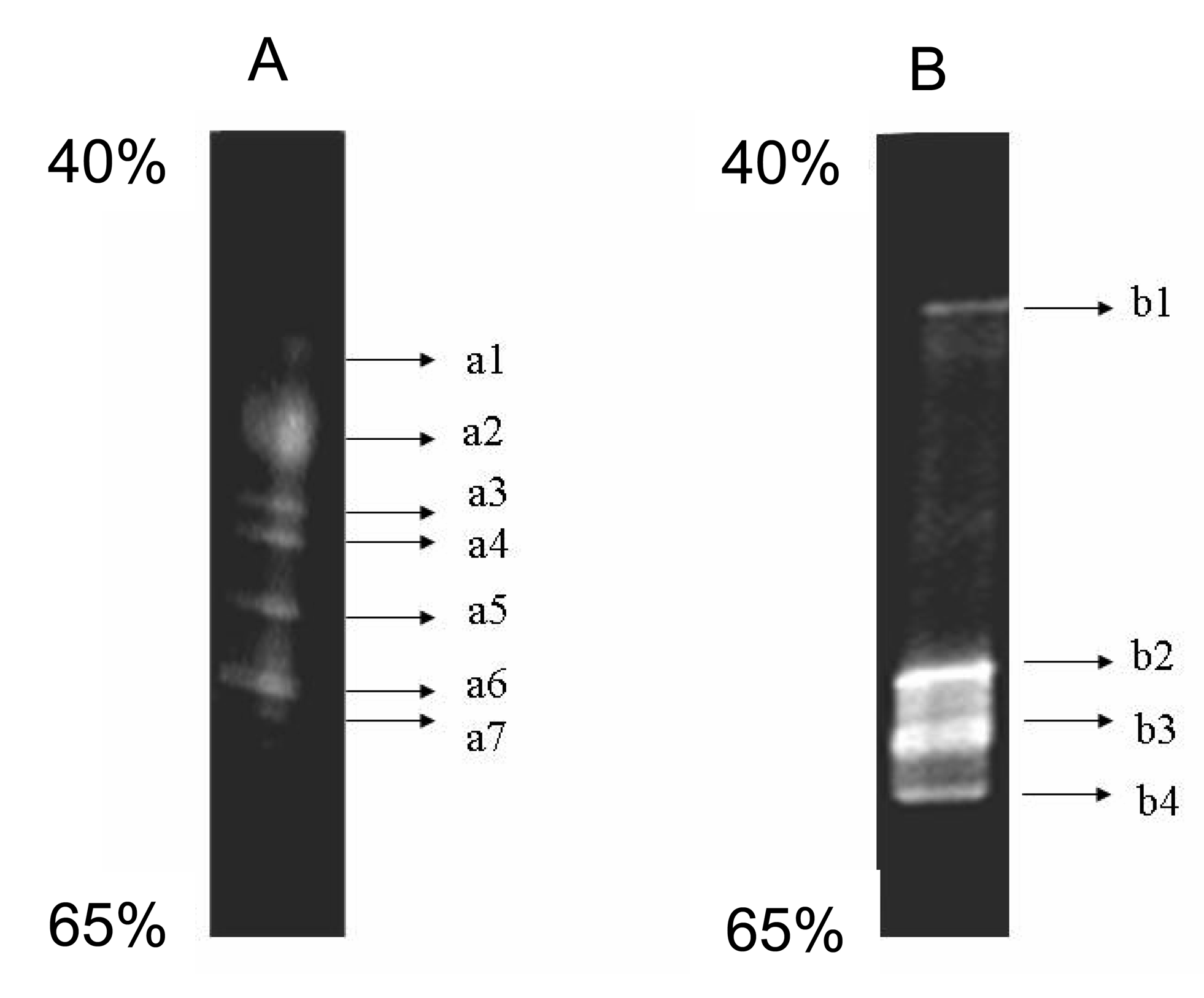

Supplement: Figure S3 — DGGE analysis of mixed biofilm consortia. DGGE analysis of DNA extracted from mixed biofilm consortia and amplified with bacterial 16S rRNA gene specific primers (f968-GC and R1401a/b) (A) or phototroph 23S rRNA gene specific primers (p23SrV_f1 and p23SrV_r1) (B). Bands were excised, purified, re-amplified using the primers f968 and R1401a/b or p23SrV_f1 p23SrV_r1 and sequenced. BLAST searches suggested Phormidium (a1, a5), Leptolyngbya, (a6, b2), Pseudanabaena (b1 and b3) and Cyanothece (b4) cyanobacteria. The chemotrophic bacteria phylogenetically most similar to Sediminibactetium (a2), Prosthecobacter (a3, a4) and Methylococcus (a7) were also detected. (0.22 MB TIF) [file pone.0010821.s003.tif]

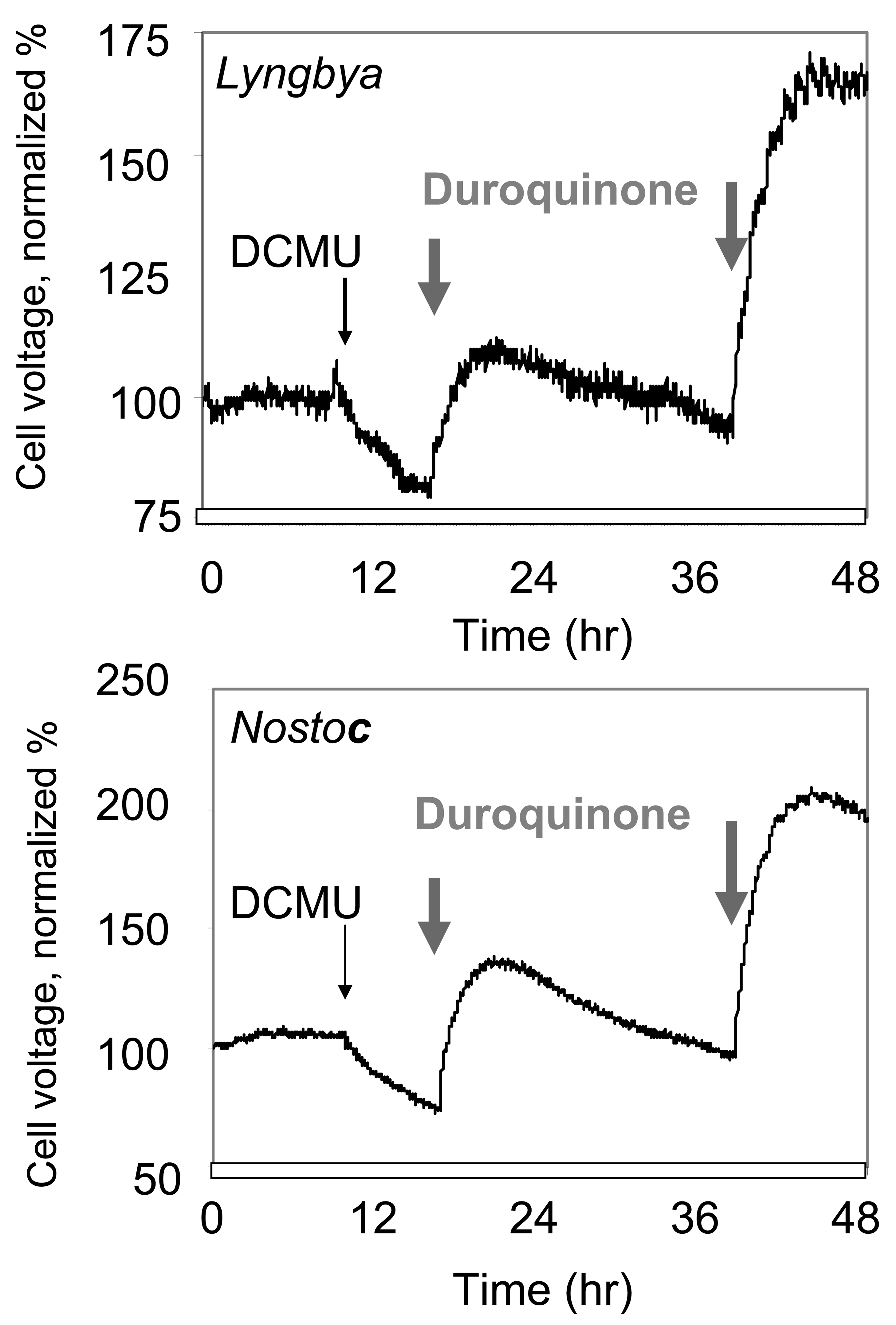

Supplement: Figure S4 — Duroquinone rescues DCMU-inhibited electrogenic activity. Administration of 10 µM DCMU (black arrows) to Lyngbya (top) or Nostoc (bottom) containing MFCs partly inhibited electrogenic activity under constant 100 lux light. After DCMU-associated inhibition of electrogenic activity reached a stable plateau, treatement with 25 µM duroquinone (grey arrows) fully restored electrogenic. Subsequent treatment with additional 75 µM duroquinone (second grey arrows) boosted the electrogenic activity. (0.41 MB TIF) [file pone.0010821.s004.tif]
