# Supplementary material for: Light-Dependent Electrogenic Activity of Cyanobacteria
Source: PLoS One. 2010 May 25;5(5):e10821. doi: 10.1371/journal.pone.0010821 (PMC2876029; doi:10.1371/journal.pone.0010821)
Supplement: Text S1 — (0.04 MB DOC) [file pone.0010821.s005.doc]

**Supporting Information**

**Light-dependent electrogenic activity of cyanobacteria**

John M. Pisciotta, Yongjin Zou, Ilia V. Baskakov

Center for Biomedical Engineering and Technology, University of Maryland, Baltimore, MD 21201, USA

**Table S1.** DGGE Primers

| Primer Set | Gene | Specificity |
| --- | --- | --- |
| 1401 | 16S | Eubacteria [1] |
| 50f * | 16S | Cyanobacteria [2] |
| 23SrV ** | 23S | Cyanobacteria +Algae [3] |

f968_GC CGC CCG GGG CGC GCC CCG GGC GGG GCG GGG GCA CGG GGG GAA CGC GAA GAA CCT TAC

f968 GGA CAG AAA GAC CCT ATG AA

r1401a/b TCA GCC TGT TAT CCC TAG AG

50f_GC CGC CCG GGG CGC GCC CCG GGC GGG GCG GGG GCA CGG GGG GAA CAC ATG CAA GTC GAA CG

50f AAC ACA TGC AAG TCG AAC G

r781a GAC TAC TGG GGT ATC TAA TCC CAT T

* 50S Primers and conditions adapted from Nubel and coauthors [2].

p23SrV_f1_GC CGC CCG GGG CGC GCC CCG GGC GGG GCG GGG GCA CGG GGG GGG ACA GAA AGA CCC TAT GAA

p23SrV_f1 GGA CAG AAA GAC CCT ATG AA

p23SrV_r1 TCA GCC TGT TAT CCC TAG AG

** 23S Primers and conditions adapted from Sherwood and Presting [3] for DGGE by the addition of a GC clamp to the p23SrV_f1 primer.

Reference List

1. Brons JK, van Elsas JD (2008) Analysis of bacterial communities in soil by use of denaturing gradient gel electrophoresis and clone libraries, as influenced by different reverse primers. Appl Environ Microbiol 74: 2717-2727.

2. Nübel U, Garcia-Pichel F, Muyzer G (1997) PCR primers to amplify 16S rRNA genes from cyanobacteria. Appl Environ Microbiol 63: 3327-3332.

3. Sherwood A, Presting G (2007) Universal primers amplify a 23S rDNA plastid marker in eukaryotic algae and cyanobacteria. J Phycol 43: 605-608.

4. Qiao Y, Li CM, Bao SJ, Lu Z, Hong Y (2008) Direct electrochemistry and electrocatalytic mechanism of evolved *Escherichia coli* cells in microbial fuel cells. Chem Commun (Camb) 21: 1290-1292.

5. Sharma T, Reddy AL, Chandra TS, Ramaprabhu S (2008) High power density from Pt thin film electrodes based microbial fuel cell. J Nanosci Nanotechnol 8: 4132-4134.
